# Metastatic High-Grade Endometrial Stromal Sarcoma Presenting With Recurrent Bilateral Pneumothoraces

**DOI:** 10.7759/cureus.106717

**Published:** 2026-04-09

**Authors:** Hana Chakalian, Amna Asif, Amanda Frugoli, Colin Scibetta, Robert Bernstein

**Affiliations:** 1 Graduate Medical Education, Community Memorial Healthcare, Ventura, USA; 2 Internal Medicine, Community Memorial Healthcare, Ventura, USA; 3 Internal Medicine, Free Spirit Health and Healing Inc, Ventura, USA; 4 Pulmonary Medicine, Community Memorial Healthcare, Ventura, USA

**Keywords:** catamenial pneumothorax, endometrial stromal sarcomas, pneumothorax from a non-bronchogenic extrathoracic source, pulmonary metastases, recurrent bilateral pneumothorax

## Abstract

Endometrial stromal sarcoma is a rare malignancy that can be difficult to diagnose due to its nonspecific presentation. Unfortunately, the presenting symptoms of abnormal uterine bleeding (AUB), anemia, and pelvic pain overlap with symptoms of benign uterine fibroids, which can delay diagnosis. Additionally, initial evaluations with imaging or even a negative endometrial biopsy may provide false reassurance. Due to novelty, mimicry of benign uterine processes, and high false-negative endometrial biopsy rates, this malignancy is often diagnosed at advanced stages. In this case vignette, we describe a case of metastatic high-grade endometrial stromal sarcoma (HG-ESS) presenting with menorrhagia, pulmonary embolism, and recurrent pneumothoraces in a 42-year-old G1P1 woman.

## Introduction

Uterine tumors have an important impact on women’s health. Benign uterine tumors such as fibroids have the highest incidence of any tumor in women globally [1,2]. Unfortunately, the presenting symptoms of these tumors, which include abnormal uterine bleeding (AUB), anemia, and pelvic pain, overlap with symptoms of uterine malignancy [2-4]. This can delay the diagnosis of an aggressive and often terminal disease [3]. AUB can be used to describe heavy bleeding with menstruation, abnormal frequency of uterine bleeding, and prolonged uterine bleeding [1,2]. Women who have completed menopause but have uterine bleeding are typically considered to have postmenopausal bleeding [1,2]. Though leiomyomas are not known to transform into cancerous lesions, endometrial sarcomas can be mistaken for these benign tumors. This is due to the lack of sensitive diagnostic techniques and their resemblance to normal, proliferating endometrial cells [4]. Endometrial stromal sarcomas (ESS) are rare, and early detection necessitates a high index of suspicion. Diagnostic modalities include transvaginal ultrasound, endometrial aspiration cytology with office-based endometrial biopsy, dilatation and curettage, and hysterectomy [5]. Clinicians often turn to pipelle endometrial biopsies as a first step to diagnosing a suspected cancer of the endometrium. However, in ESS, a pipelle biopsy has only 50% sensitivity, making it a relatively poor diagnostic tool [6].

ESS is further classified into four sub-categories, including endometrial stromal nodules, low-grade ESS (LG-ESS), high-grade ESS (HG-ESS), and undifferentiated uterine sarcoma [7]. Classification is based on histopathology, using muscle infiltration and the number of mitoses per high-power field [7]. High-grade endometrial sarcomas have a median progression-free survival (PFS) of 7-11 months [8]. Low-grade endometrial sarcomas have a more optimistic prognosis with a five-year survival rate of 89.3% in stage I-II tumors and a 50.3% rate in stage IV [8,9]. Though the course of ESS is typically described as indolent, metastasis can occur [4,9]. The lungs and abdomen are the most common extra-pelvic sites of metastasis [3,4]. Pulmonary metastatic lesions can be single or multiple and can result in spontaneous pneumothorax [9]. Additionally, there are case reports of uterine sarcoma extra-pelvic metastasis presenting as spontaneous pneumothorax [9].

In this case vignette, we describe a case of metastatic HG-ESS presenting with menorrhagia, pulmonary embolism, and recurrent pneumothoraces. This case highlights the difficulty in diagnosing this rare malignancy and the need for high clinical suspicion.

## Case presentation

**Figure 1:**
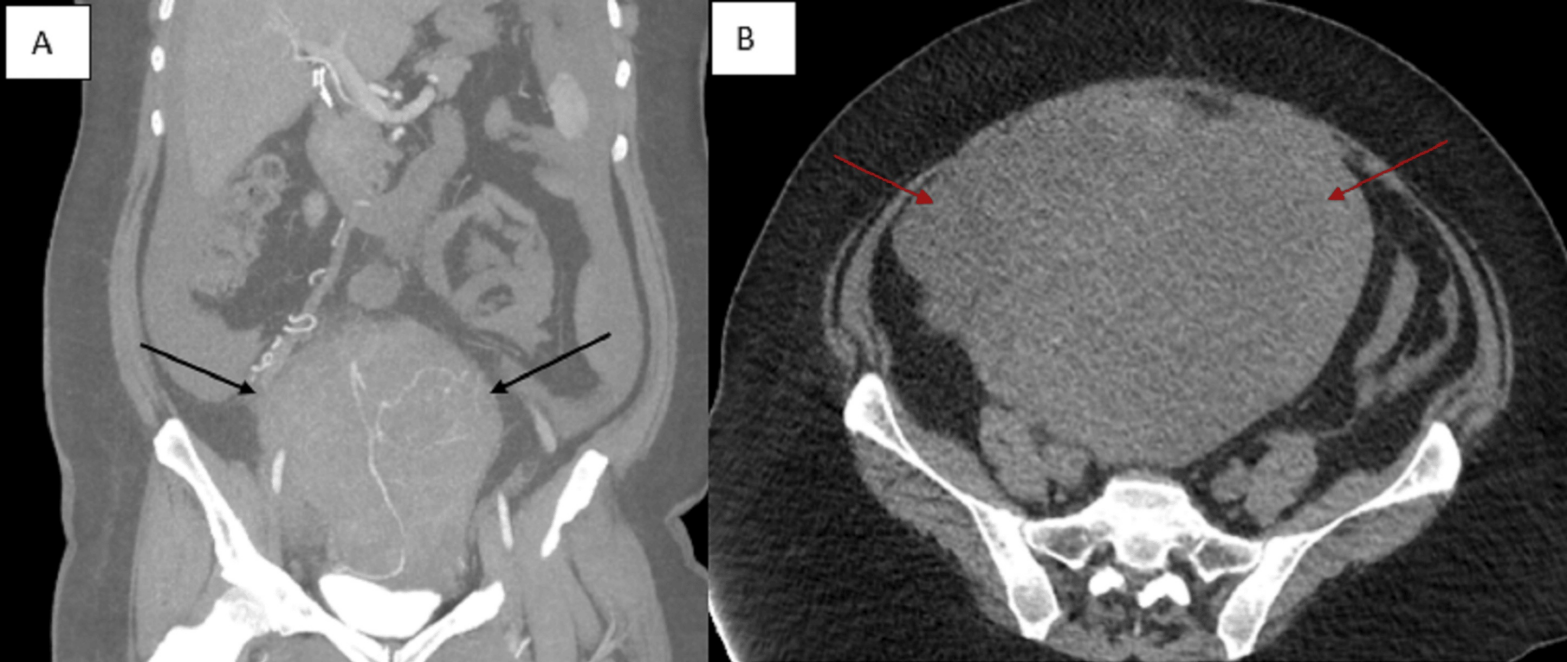
Contrast-enhanced computed tomography of the abdomen and pelvis. (A) Sagittal view with enlarged uterus (black arrows); (B) coronal view with enlarged nodular uterus (red arrows)

A 42-year-old (G1P1) woman with a past medical history of obesity and uterine fibroids with associated menorrhagia for two years presented to the hospital for an elective hysterectomy. Outpatient imaging included transvaginal ultrasound and computed tomography (CT) abdomen and pelvis, with and without contrast. CT with IV contrast demonstrated an enlarged uterus measuring 14.5 × 16.4 × 16.4 cm with a lobulated appearance. An ill-defined area of low attenuation measuring 10.8 × 10.5 × 9 cm was also visualized (Figure 1). Pipelle endometrial biopsy performed one month prior was negative for malignancy.

**Figure 2:**
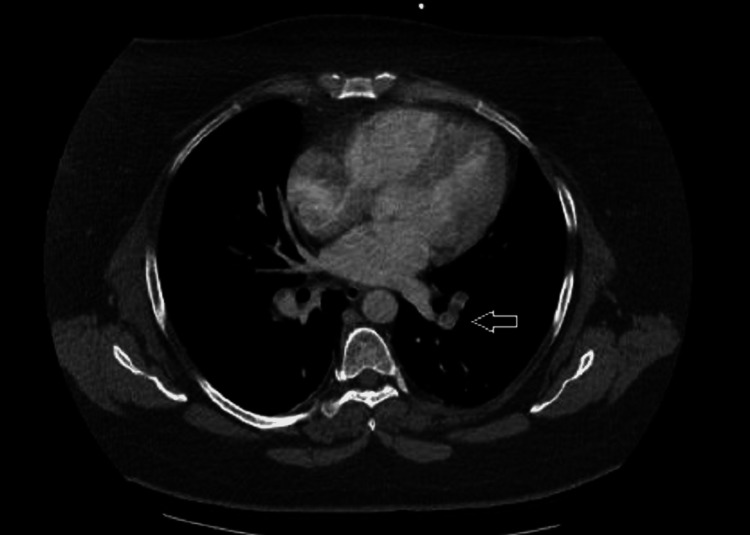
CT chest angiogram demonstrating bilateral pulmonary embolism (white arrow) CT: computed tomography

In the operating room, the patient developed acute hypoxia and tachycardia, prompting immediate cessation of surgery. Evaluation for acute hypoxia revealed multiple pulmonary emboli on a CT chest angiogram (Figure 2). The patient did report several relatives with a history of blood clots, which raised suspicion for an underlying clotting disorder. Surgery was deferred in favor of hypercoagulable workup, and the patient was discharged on apixaban. 

**Figure 3:**
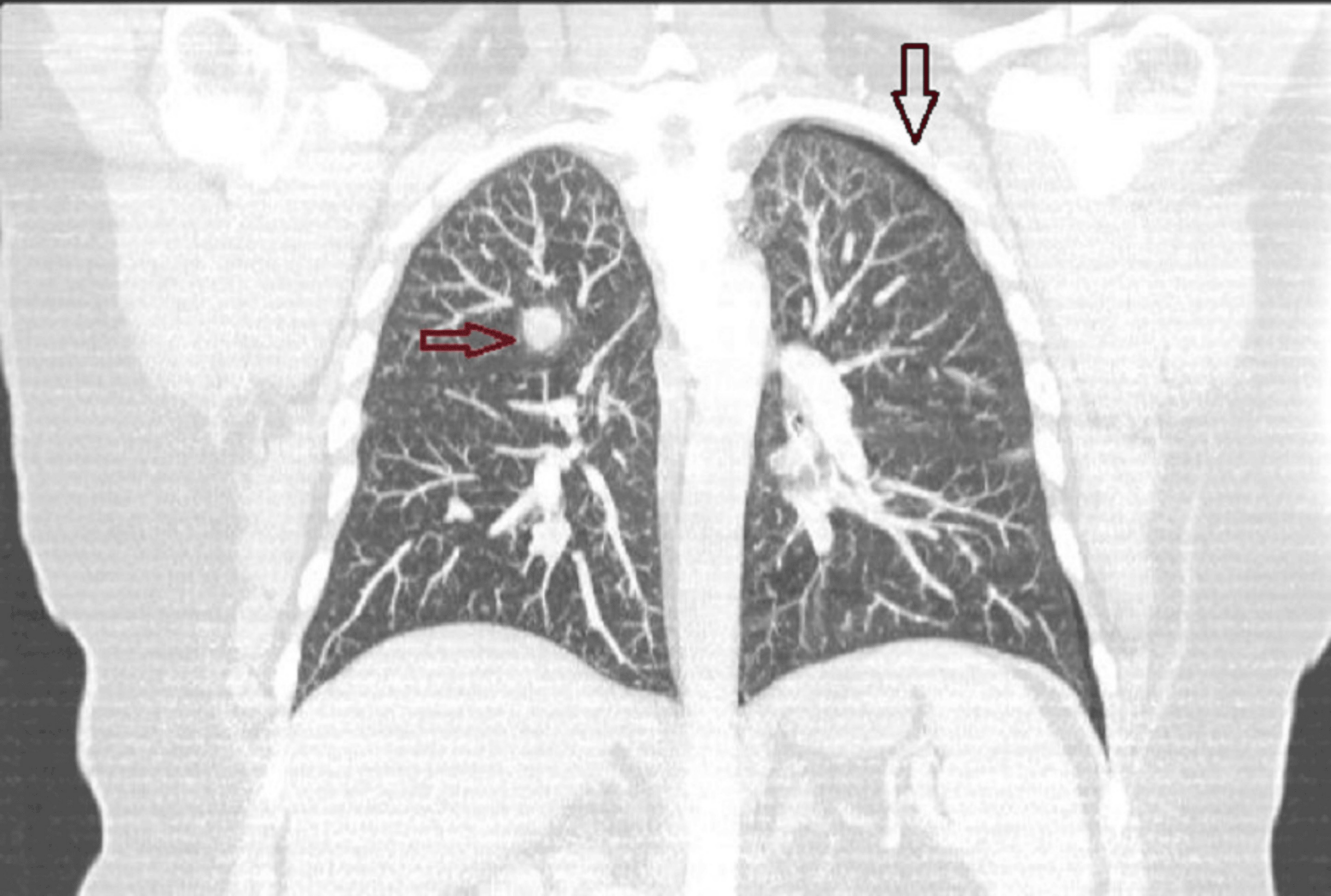
CT chest angiogram demonstrating right nodular mass (red arrow) and left small pneumothorax (black arrow) CT: computed tomography

Approximately one month later, she presented to the hospital with worsening dyspnea, chest pain, and cough without recent trauma or travel. Repeat CT chest angiogram ruled out recurrence of pulmonary embolism or pulmonary infarct. A small, left apical pneumothorax, in addition to approximately six lung nodules measuring from 1 to 2 cm throughout all lung lobes, was present and not previously observed (Figure 3).

**Figure 4:**
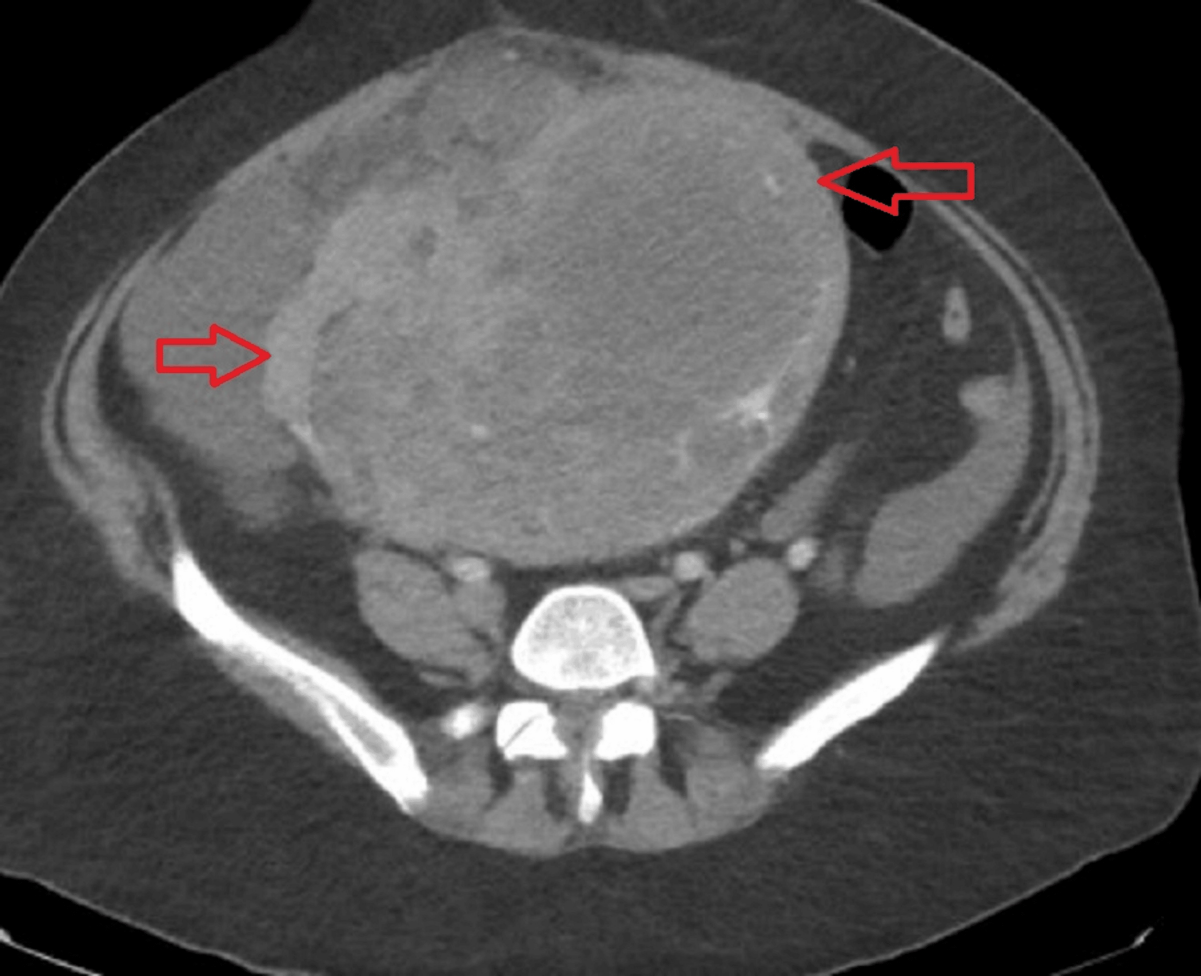
CT abdomen and pelvis with IV contrast demonstrating an enlarging uterus with a heterogeneous mass (red arrows). Specifically, the uterus has increased by 10 cm in 2 months CT: computed tomography

A 10-French chest tube was placed, but the pneumothorax progressed, requiring placement of two additional 20-French chest tubes. Infectious work-up was negative for coccidioidomycosis, tuberculosis, SARS-CoV-2, and cryptococcal infection. Throughout her hospitalization, she continued to experience menorrhagia with a precipitous change in her hemoglobin levels down to 6.1, necessitating multiple blood transfusions. This was suspected to be related to uterine tumor bleeding while on anticoagulation. CT of the abdomen and pelvis with contrast demonstrated a large mass-like-appearing uterus, which was thought to represent sequelae of multiple uterine fibroids with necrosis. Notably, the uterus was found to be 10 cm larger than at the prior examination (Figure 4).

Hysterectomy was planned following discharge and stabilization of pneumothorax, as the two issues were suspected to be unrelated during the early part of the patient's presentation. Malignancy was considered an unlikely cause of the nodules, given the appearance of new and regressing nodules since initial hospitalization and a negative endometrial biopsy prior to surgery.

**Figure 5:**
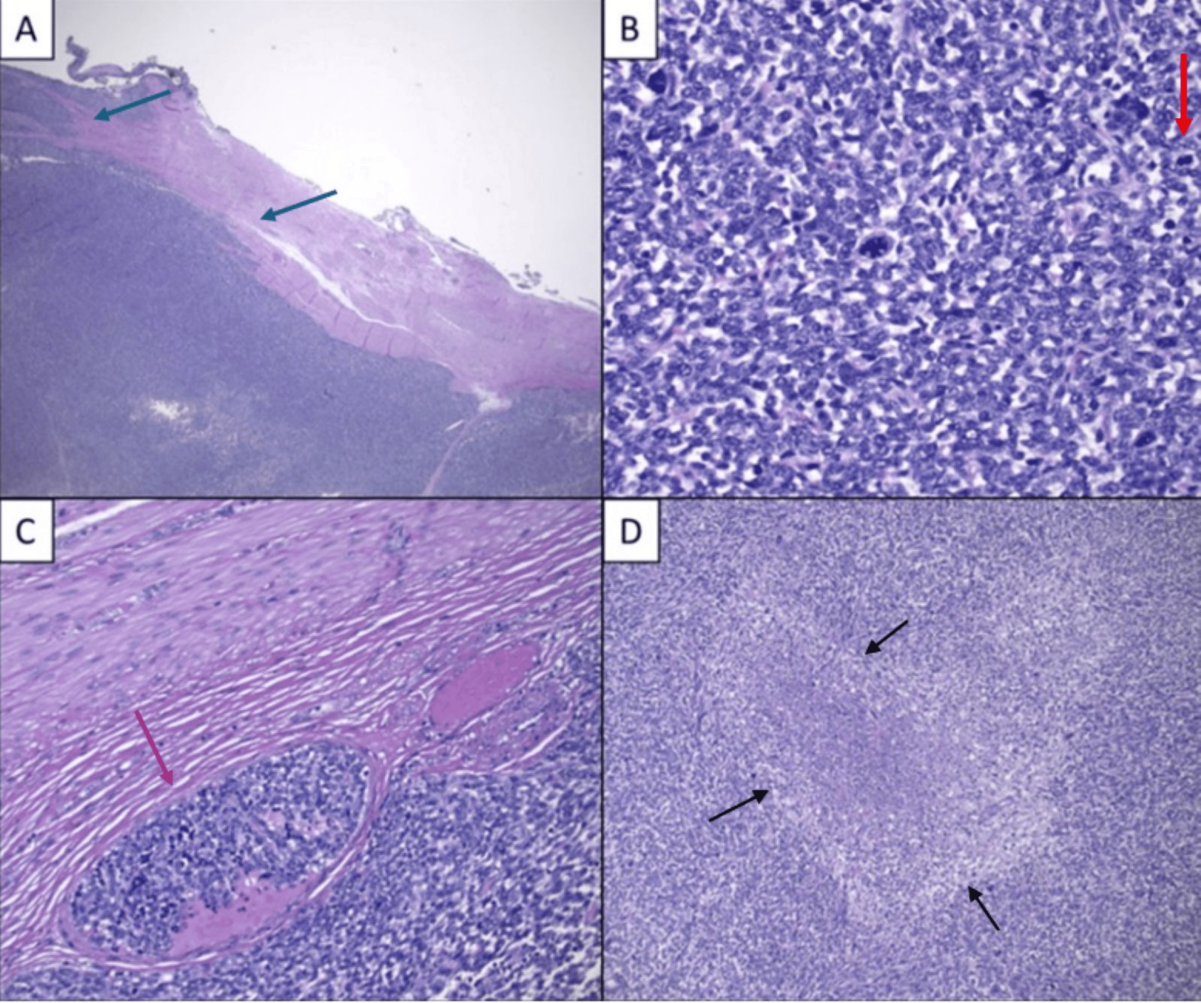
Hematoxylin and eosin (H&E) staining demonstrating high-grade endometrial stromal sarcoma. (A) Infiltrating myometrium and involving the serosa of the uterus (blue arrow) (20×); (B) numerous mitotic figures (>10/10 high-power fields (HPF)) including atypical mitoses (red arrow) (400×); (C) involvement of adjacent blood vessels (purple arrow) (200×); (D) tumor necrosis (black arrow) (200×) Image (A) with infiltrating myometrium supports the diagnosis of uterine sarcoma over stromal myosis. Image (B) demonstrates a high mitotic rate, with ≥10 mitoses per 10 HPF, indicating stromal sarcoma. Image (C) with invasion of blood vessels supports hematologic metastasis to the lungs. Image (D) with necrosis likely contributed to the severity of uterine bleeding.

Ultimately, an emergent hysterectomy was performed as the patient became hemodynamically unstable due to profuse and persistent uterine bleeding. The uterus was noted to be adherent to the omentum, sigmoid colon, and bladder. Pathology reported the uterus measured 20 cm with a tumor measuring >5 cm eroding through the uterine wall into the right pelvic peritoneum. Surgical pathology revealed HG-ESS invading more than half of the myometrium and extending into the serosal surface and parametrium (Figures 5A-5D). HG-ESS was determined due to infiltration into the uterine wall and ≥10 mitoses per 10 high-power field.

Shortly after the surgery, the patient developed bilateral tension pneumothorax necessitating bilateral chest tube insertion. Due to a lack of resolution of pneumothorax throughout the hospital course, a video-assisted thoracoscopic surgery (VATS) with talc pleurodesis was planned. A perioperative inferior vena cava (IVC) filter was placed, and prophylactic heparin was paused for surgery. Despite intervention with VATS, she continued to have a persistent left apical pneumothorax, and a left chest tube remained in place. Biopsy of the pulmonary nodules was not successful during VATS as the patient had repeated rapid oxygen desaturations requiring lung reinflation. On visual inspection at the time of surgery, these nodules appeared to have grown, supporting the suspicion of metastatic disease. She was evaluated by gynecology-oncology and hematology-oncology specialists and underwent palliative treatment with gemcitabine and Taxotere. During her prolonged hospital stay, she received two cycles of treatment with plans to continue as an outpatient.

She was able to complete five total rounds of chemotherapy but was readmitted almost one year later due to intractable nausea and vomiting. During this admission, she was found to have tumor progression on CT with the development of omental caking. Her treatment was changed to a liposomal formulation of doxorubicin HCl, and she planned to seek a clinical trial. Despite these treatments, she expired 14 months after diagnosis (Table 1). 

**Table 1:** Timeline of clinically significant events CT: computed tomography; VATS: video-assisted thoracoscopic surgery; IVC: inferior vena cava

Month/year	Event	Notes/findings
Initial	Endometrial pipelle biopsy	Negative for malignancy
Initial	CT abdomen/pelvis	Enlarged uterus 14.5 × 16.4 × 16.4 cm; lobulated; ill-defined low attenuation 10.8 × 10.5 × 9 cm
August Year 1	Elective hysterectomy (aborted)	Procedure aborted due to hypoxia from hypotension
August Year 1	CT chest angiogram	Pulmonary embolism identified
August Year 1	Hospital admission 14 days	Pulmonary embolism treated with apixaban, complicated by uterine bleeding
August Year 1	Uterine artery ablation	Completed by interventional radiology during hospitalization with resolution of uterine bleeding
October-December Year 1	Hospital admission 63 days	Shortness of breath → left-sided pneumothorax
October-December Year 1	Imaging	CT chest: moderate left pneumothorax, multiple pulmonary nodules 2 cm; CT abdomen/pelvis: uterus 24 cm, mixed density mass, calcifications (10 cm larger than initial CT)
October-December Year 1	Interventions	Left chest tube, then bilateral chest tubes; VATS for persistent left-sided pneumothorax; IVC filter placed; Port-A-Cath placed, total abdominal hysterectomy + BSO + bilateral hypogastric artery ligation
October-December Year 1	Chemotherapy	Gemcitabine and Taxotere were started for high-grade endometrial stromal sarcoma, stage IV
December-October Year 2	Chemotherapy	Completes 5 rounds of chemotherapy
October Year 2	Hospitalization 7 days	Intractable nausea/vomiting; imaging shows omental caking and evidence of cancer progression
October Year 2	Treatment change	Starts second-line liposomal doxorubicin; declines hospice, seeks clinical trial

## Discussion

ESS are rare tumors with peak incidence in pre-menopausal women between the ages of 40 and 55 who present with AUB or pelvic pain [10]. Although ESS is very rare, lung metastasis for this specific tumor can have an incidence of 7%-28% [11,12]. One study revealed that in four out of 10 reported cases of women with ESS and pneumothoraces, the pneumothorax was the first presenting sign of the malignancy [12]. They are commonly mistaken for benign leiomyomas, as in the case above, or other benign diseases due to their close resemblance to normal proliferative endometrial cells. The American College of Obstetricians and Gynecologists (ACOG) recommends classifying the etiologies of AUB using the PALM-COEIN system [13].

Our patient underwent a gynecologic evaluation, including imaging and biopsy. Her etiology was determined to be structural, due to leiomyoma [13]. She did not respond to medical management nor to uterine artery ablation [14]. Due to ongoing AUB, a hysterectomy was planned but was delayed due to ongoing related complications. The complex presentation with metastases, including pneumothoraces and pulmonary emboli, led to delays in histological diagnosis and treatment. Although this patient had a negative endometrial biopsy prior to her diagnosis, this test has poor sensitivity for sarcoma in patients with AUB [15].

Pulmonary metastasis was not confirmed with histology but is congruent with her presentation and imaging. The patient’s thrombi on the IVC filter, despite anticoagulation and multifocal pulmonary emboli, are consistent with the hypercoagulable state associated with malignancy [16]. This assumption was later supported on an outpatient basis when PET-CT revealed nodules were hypermetabolic.

The patient’s longitudinal course, characterized by pulmonary embolism, pulmonary nodules with recurrent pneumothorax, and severe AUB, was concerning for an underlying malignancy. However, multiple contemporaneous confounding factors favored a benign etiology at the time of her presentation. A family history suggestive of a hypercoagulable disorder helped explain the pulmonary embolism, and a prior benign endometrial biopsy for AUB provided false reassurance, supporting the presumption of a more common benign process, such as leiomyoma progression. These factors collectively contributed to a delay in diagnosis.

This case highlights the diagnostic challenges associated with uterine sarcomas, given their rarity (estimated incidence of 1-2 cases per million annually) and the limited sensitivity of pipelle endometrial biopsy. Negative biopsy results provide unwarranted reassurance and can contribute to delays in diagnosis. Consequently, more than half of patients with HG-ESS present with advanced-stage disease. Similar confounding clinical features, as demonstrated in this case, may contribute to delayed recognition.

## Conclusions

We present this case to raise awareness among other physicians/providers and improve the diagnosis of this rare disease. This case vignette highlights the aggressive progression of HG-ESS. This case also outlines the diagnostic complexity. A high index of suspicion is needed to differentiate uterine sarcoma and other uterine malignancies from leiomyoma. Ongoing complications such as recurrent pneumothoraces and thrombosis, and the setting of menorrhagia and fibroids should prompt consideration for endometrial malignancy. Physicians should be aware of the high false-negative rate of endometrial biopsy. Further investigation and consideration of hysterectomy should be undertaken in refractory menorrhagia and persistent symptoms.
